# Supplementary material for: Genetic gains underpinning a little-known strawberry Green Revolution
Source: Nat Commun. 2024 Mar 19;15:2468. doi: 10.1038/s41467-024-46421-6 (PMC10951273; doi:10.1038/s41467-024-46421-6)
Supplement: Supplementary file 1 — Supplementary Information [file 41467_2024_46421_MOESM1_ESM.pdf]

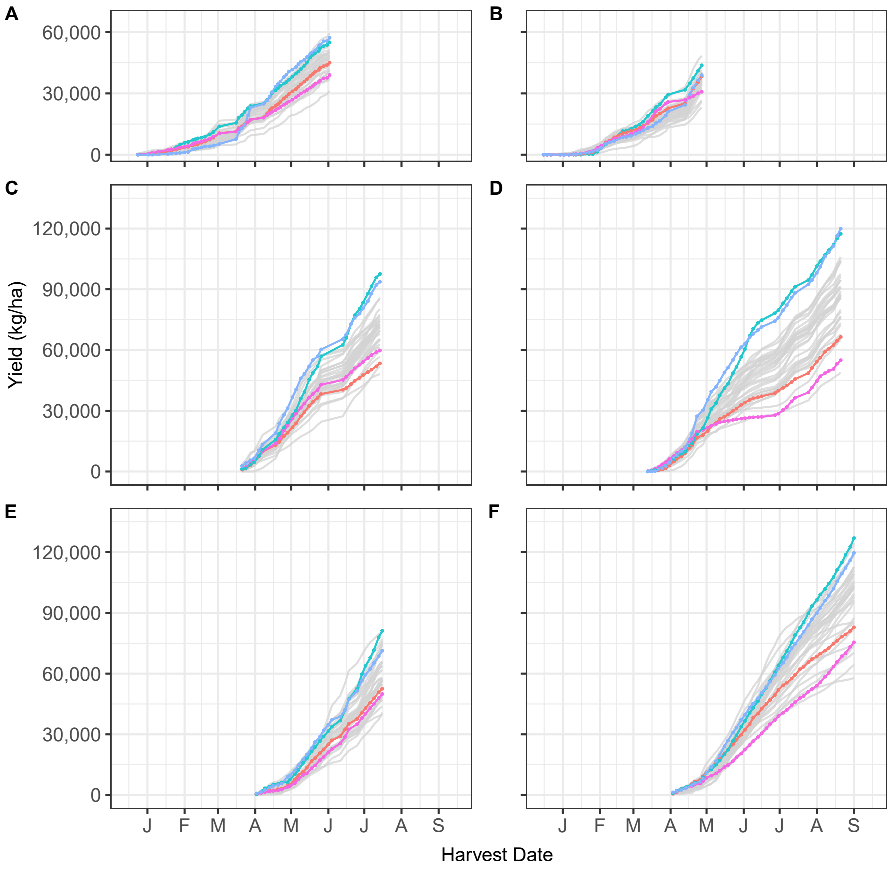

Supplementary Figure 1. Cumulative marketable fruit yields of modern short-day and day-neutral cultivars (hybrids) grown on coastal California farms over the 2015-16 and 2016-17 growing seasons. Estimated marginal means are displayed for 46 to 52 fruit harvests from 31 short-day hybrids grown in Oxnard, CA (A-B) and 33 day-neutral hybrids grown in Santa Maria, CA (C-D) and Prunedale, CA (E-F). (A, C, and E) The plots shown in the left column display yield EMMs from the 2016 harvest season (January to September on the x-axes). (B, D, and F) The plots shown in the right column display yield EMMs from the 2017 harvest season. (A-B) The color highlighted short-day cultivars in the upper row are 'Fronteras' (teal), 'UCD Victor' (light blue), 'Grenada' (salmon), and 'Petaluma' (pink). (C-F) The color highlighted day-neutral cultivars in the middle and lower rows are 'UCD Royal Royce' (teal), 'UCD Valiant' (light blue), 'Monterey' (salmon), and 'San Andreas' (pink).

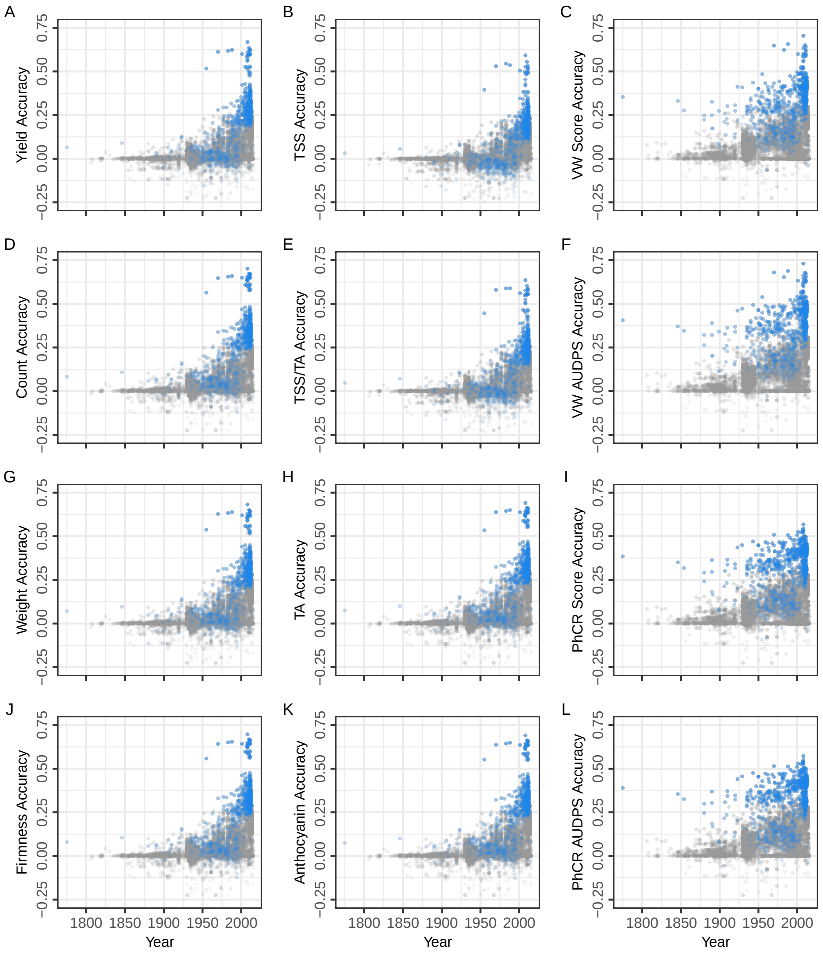

Supplementary Figure 2. Accuracy of genomic-estimated breeding values (GEBVs) estimated by ss-BLUP for agriculturally important traits among hybrids spanning the domestication history of strawberry (1775-present). GEBVs were estimated for 6,419 hybrids with known birth years (1775-2015) using ss-BLUP. ss-BLUP GEBV accuracies were estimated for every hybrid by $1 - \sqrt{PEV_i/\hat{\sigma}^2_A}$, where PEV_i is the prediction error variance of the i-th hybrid and σ^2_A is the additive genetic variance. (A, D, G, and J) Statistics are shown in the left hand column of plots for fruit yield , count, weight, and firmness. (B, E, H, and K) Abbreviations for fruit quality traits shown in the center column of plots are TSS = total soluble solids and TA = titratable acidity. (C, F, I, and L) Abbreviations for disease resistance traits shown in the right hand column of plots are Verticillium wilt (VW) resistance score and area under the disease pressure stairs (AUDPS) and Phytophthora crown rot (PhCR) resistance score and AUDPS.

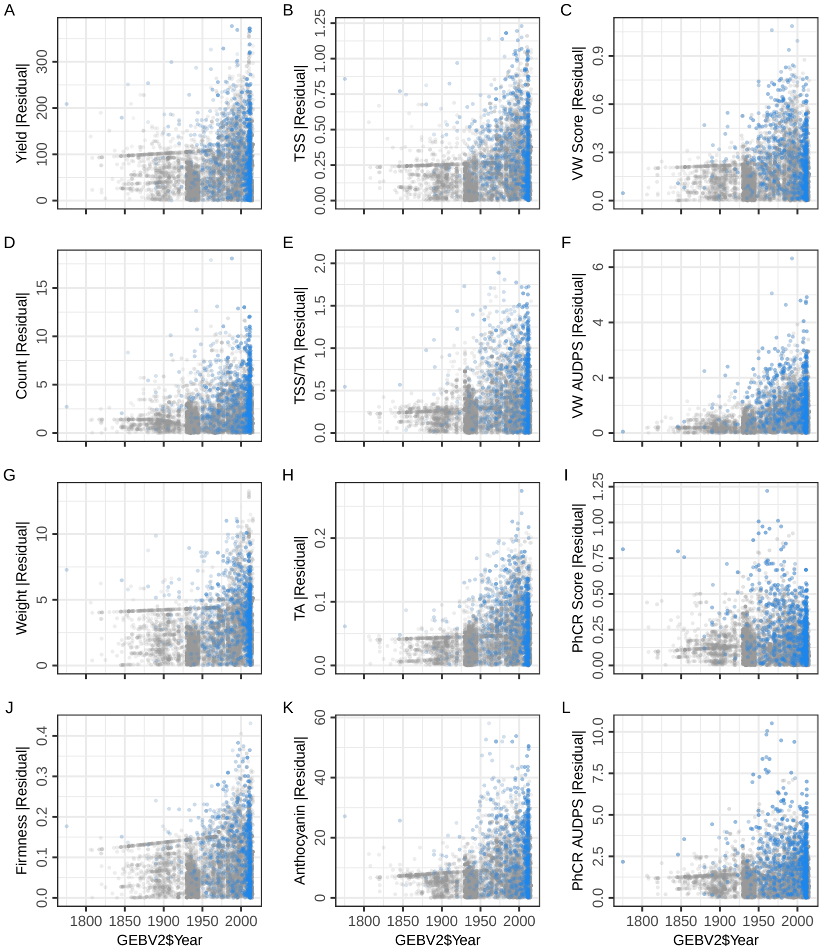

Supplementary Figure 3. Transformed residuals (differences between observed and predicted GEBVs) from piecewise linear regressions of genomic-estimated breeding values (GEBVs) on birth years for agriculturally important traits among hybrids spanning the domestication history of strawberry (1775-present). GEBVs were estimated for 6,419 hybrids with known birth years (1775-2015) using ss-BLUP. The residuals were transformed by $\sqrt{(y-\hat{y})^2}$, where y is the GEBV for a hybrid and $\hat{y}$ is the predicted GEBV mean for a birth year. The dashed lines depict the predicted GEBV means from piecewise linear regressions of GEBVs on birth years before and after estimated change-point (CP) years (1775 to CP and CP to 2015). (A, D, G, and J) Statistics are shown in the left hand column of plots for fruit yield , count, weight, and firmness. (B, E, H, and K) Abbreviations for fruit quality traits shown in the center column of plots are TSS = total soluble solids and TA = titratable acidity. (C, F, I, and L) Abbreviations for disease resistance traits shown in the right hand column of plots are Verticillium wilt (VW) resistance score and area under the disease pressure stairs (AUDPS) and Phytophthora crown rot (PhCR) resistance score and AUDPS.

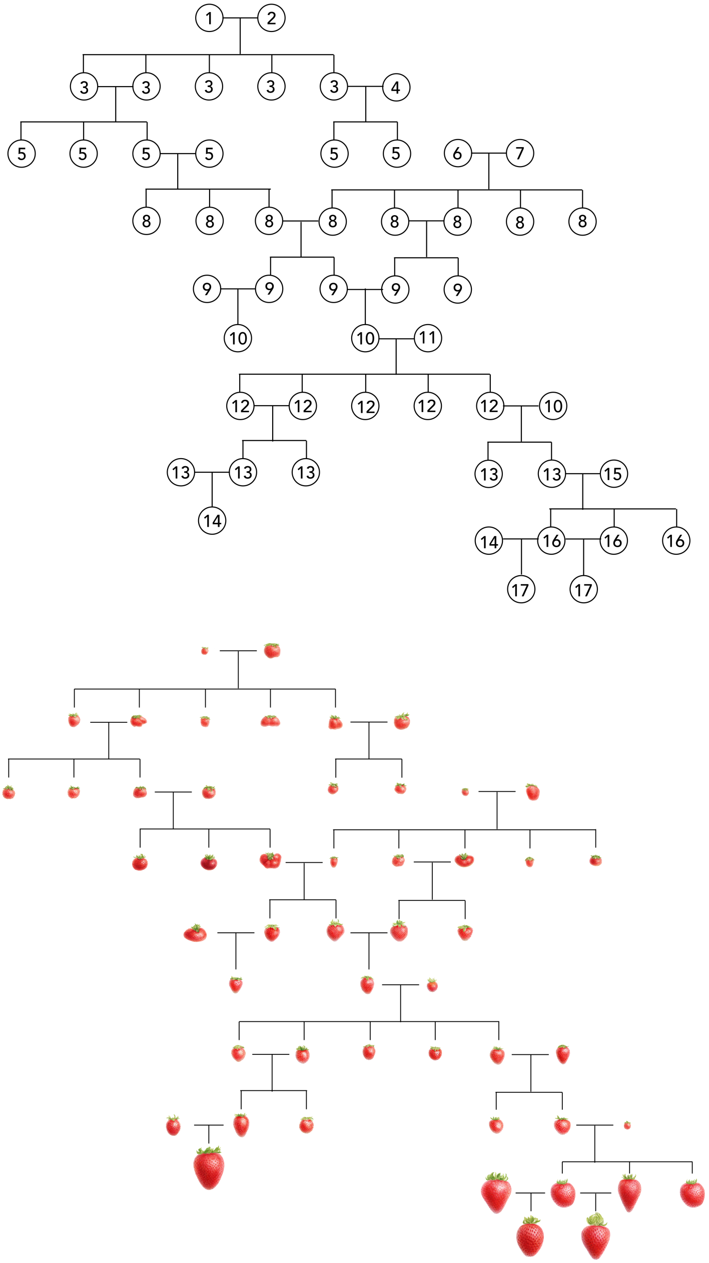

Supplementary Figure 4. Catalog of the botanical drawings, photographs, and other information used to illustrate the fruit morphology changes shown in Figure 4. The images shown are 51 original watercolors developed from botanical illustrations of extinct ancestors and photographs of living specimens by Sandra Doyle© (https://www.sandra-doyle.co.uk/; commissioned by UC Davis and used with permission of the artist). The numbers shown in the upper panel identify the footnotes enumerated in Supplementary Data 15. See the Figure 4 caption for additional details.
